# Weight distribution asymmetry in relation to walking speed in children with spastic cerebral palsy

**DOI:** 10.4314/ahs.v22i2.65

**Published:** 2022-06

**Authors:** Nahla M Ibrahim, Mai Elsayed Abbass

**Affiliations:** Department of Physical Therapy for Paediatrics, Faculty of Physical Therapy, Cairo University, Giza, Egypt

**Keywords:** Physical Therapy Modalities, Pediatrics, Rehabilitation

## Abstract

**Background:**

Gait speed and postural stability are indicators of community level ambulation and may be a valuable measure of disability.

**Objectives:**

to investigate the relation between the distribution of weight on both lower extremities and gait speed in children with spastic cerebral palsy.

**Methods:**

Evaluation for weight distribution on both lower limbs and speed during gait for sixty children with spastic diplegia and forty-five children with hemiplegia was carried out by the Biodex gait trainer. Pearson correlation test was conducted to determine the relation of the symmetry index and the percent of weight bearing to speed.

**Results:**

A significant weak positive correlation was found between speed and symmetry index in diplegic group, while there was a non-significant weak negative correlation between speed and symmetry index was noticed in hemiplegic group. Nonsignificant weak positive correlation between speed and weight on most affected side was recorded in diplegic group. While in hemiplegic group, there was significant weak negative correlation between weight on affected side and speed.

**Conclusion:**

Children with cerebral palsy demonstrate asymmetrical weight distribution during walking. Physical therapy training should be directed to enhance weight bearing distribution thus improving gait and postural stability.

## Introduction

Children with Cerebral Palsy (CP) show a different walking pattern than their normal peers as they tend to slow their walking speed to ensure more stability^1^,^2^. They attempt to increase their step width to be able to keep lateral balance and they also decrease the step length to improve the balance in anteroposterior directions 3,4. Increasing the step width causes asymmetry in weight distribution on both lower limbs during gait that seems to stress less on the affected side that causes increased energy expenditure 5.

Weight distribution asymmetry and unequal balance on both lower limbs during gait are often the most observed gait characteristics in hemiplegic children. In this asymmetry majority of the body weight is loaded on the non-involved lower limb with less ability to transfer their weight on the affected side 6. The comparison between uni and bilateral limb support duration as well as the percentage of stance and swing phase cycles of both lower extremities are used to detect asymmetry of gait in those children which helps to determine the ability to transfer weight between both lower limbs during gait 7.

Deviations of gait are recognized in spastic diplegic children, although most of them can walk independently. They have inequal weight distribution on lower limbs as they have a more affected and a less affected limb 8.

A vital sign in the gait rehabilitation field is the gait speed^9^. Reduced gait speed is a major problem of spastic cerebral palsy children as it causes mobility restrictions which may limit a child's abilities to participate actively and socially in daily activities and so affecting his or her quality of life 10,11. Evaluation of gait speed is of a great value in predicting the impact of disability on ambulation. Decreased speed and gait abnormalities in spastic children affect their active participation in the community and cause low quality of life. Therefore, one of the most important goals in management of gait deviations in children with CP is to accommodate walking speed to the normal level to help them participate more in the society. Gait speed is a valuable measure of disability providing a good prediction to the level of community ambulation. Improving gait speed is a major physical therapy goal as it affects participation and quality of life 12,13.

In children with cerebral palsy, postural asymmetry analysis during gait can provide information on walking control and balance, and it may help clinicians in making treatment decisions. Human gait is a continuous state of imbalance caused by the relation between center of mass (COM) and the center of pressure (COP). Every individual must maintain postural balance throughout gait 14. The aim of this study is to determine the relation between the distribution of weight on both lower extremities and speed of gait in children with spastic CP.

## Material and methods

### Sample size

Before the commencement of the study, calculation of sample size was done to avoid type II error using G*POWER statistical software (version 3.1.9.2; Franz Faul, Universitat Kiel, Germany) [Exact tests- correlational study, α=0.05, β=0.2, and moderate effect size = 0.36]. Results of the power test assumed that the appropriate sample for this correlation study is nearly sixty participants (N=60) for hemiplegic and diplegic children.

### Study Design

This research is a single, cross-sectional study conducted on spastic cerebral palsy children (60 diplegia and 54 hemiplegia). The purpose and procedure of the research was clarified to the parents of all children. All parents signed a written consent for participation of their children in the examination procedure. The project of the research was approved by the ethical committee center at the Faculty of Physical Therapy, Cairo university with number (P.T.REC/012/002973) assessment was conducted in the gait lab at Faculty of Physical Therapy, Cairo University during the period from November to December 2020.

This study was registered at clinical trials.gov with number (identifier: NCT04636424).

### Subjects

The sample of the study was sixty children with diplegia (27 boys and 33 girls) and fifty-four children with hemiplegia (24 boys and 30 girls) their age ranged from 10 to 12 years. They were selected from the pediatric outpatient physical therapy clinic. The inclusion criteria were as follows: children diagnosed as spastic diplegia or hemiplegia; the ability to stand independently without support for not less than 30 seconds; the ability to walk independently without using lower limb orthoses (hip, knee or ankle orthoses); all children were on level II according to the Gross Motor Function Classification System (GMFCS)^15^ and had the capability to understand simple verbal commands. Participants were excluded if they were using assistive devices during walking (such as canes or crutches), exposed to lower limbs orthopedic surgery or injection of botulinum toxins in the last 6 months before the evaluation, also children with fixed lower limb deformities.

Assessment was done for speed and weight distribution on both lower limbs (affected, non-affected or less affected) during gait by the Biodex gait trainer 3 (model number: 950-406, BIODEX MEDICAL SYSTEMS, INC.) It provides an objective assessment for the average right and left step length, the percentage of weight distribution on each lower limb and the average speed during gait 16. It is a treadmill with monitoring screen that allows following and recording of a person gait kinematics objectively. The parameters include: walking speed and foot-to-foot weight distribution during gait (weight distribution symmetry). Sensitive touch screen display is connected to the treadmill which allows adjustment of the examination setting. Storage of data is available and a printout is afforded for each participant evaluation that make it easy to find out the records at any time 16. Symmetry index for hemiplegic children was calculated by dividing the value of weight bearing on the affected side / the value of weight bearing on the non-affected side, while in diplegic children the symmetry index was calculated by dividing the value of the weight bearing on the most affected side by the weight bearing on the less affected side.

### Procedures for evaluation

Prior to gait evaluation, the children were first let to get familiar with the device by giving each child the chance to stand, adjust his position and decrease his tension before starting the recording of gait parameters. Every child was asked to walk over the treadmill with his comfortable speed for three to five minutes till he became adapted with the apparatus. The recording did not start until the child was comfortable and walk freely. Recording of gait parameters then started for three minutes while looking forward at the display screen, and the speed of belt was increased gradually to the limits of tolerance limit of each child. The results of gait kinematics were displayed on the screen including the speed and weight distribution on right and left lower limbs. Evaluation was repeated with a rest period in between and the average of 3 trials was recorded for each parameter.

### Statistical analysis

Descriptive statistics were utilized in presenting the subjects demographic and clinical data. Mean and standard deviation were used to summarize the quantitative variables while frequencies and percentage were used to conclude the categorical variables. Pearson correlation test was conducted to determine the relation between symmetry index, percent of weight bearing and speed. The level of significance for all statistical tests was set at p < 0.05. Statistical package for social sciences (SPSS) version 25 for windows was used to perform all the statistical measures.

## Results

### Subjects' characteristics

Sixty children with diplegia (33 girls, 27 boys) and fifty-four children with hemiplegia (30 girls,24 boys) participated in this study. Twenty-four (44.4%) children were with right hemiplegia and thirty (55.6%) were with left hemiplegia. The mean and standard deviations for age (years), weight (kilogram) and height (centimeter) in children with diplegia was (10.85 ± 0.76), (21.79 ± 1.02) and (137.26 ± 1.04) respectively, while in children with hemiplegia was (10.66 ± 0.77), (21.66 ± 1.11) and (137.16 ± 1.17) respectively.

### Symmetry index, speed and weight distribution in both groups.

The mean ± SD symmetry index in children with diplegia was 0.51 ± 0.2 and that in children with hemiplegia was 0.39 ± 0.27. The mean ± SD speed in children with diplegia was 0.42 ± 0.07 while that in children with hemiplegia was 0.50 ± 0.10. The mean weight on most affected limb and less affected limb in children with diplegia were 32.75 ± 9.37 and 67.41 ± 9.56 respectively. The mean weight on affected limb and sound limb in children with hemiplegia were 26.03 ± 13.57 and 74.1 ± 13.58 respectively.

Correlation between speed and symmetry index and weight distribution in the both groups.

### Children with diplegia

**Figure 1:**
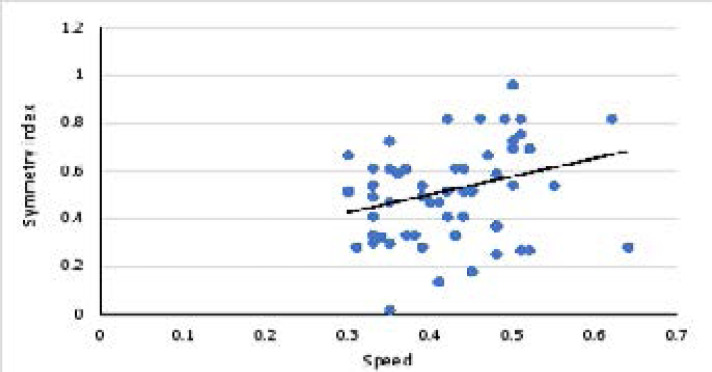
Correlation between speed and symmetry index in children with diplegia.

**Figure 2:**
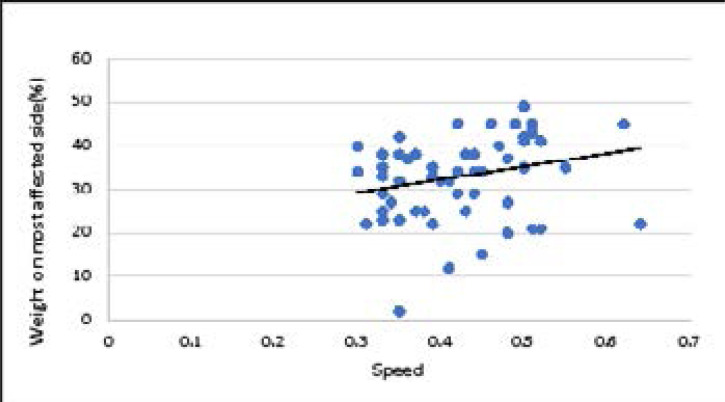
Correlation between speed and weight on most affected side in children with diplegia.

There was a significant weak positive correlation between speed and symmetry index in children with diplegia. There was a significant weak negative correlation between weight on less affected side and speed and a non-significant weak positive correlation between speed and weight on most affected side. Correlation between speed and symmetry index in children with diplegia is represented in figure (1). Correlation between speed and weight on the most affected side in children with diplegia is represented in figure (2). Table (1) represents the correlation and regression equations for diplegia.

**Table 1:** Correlation and regression equations for diplegia

		r value	p value	Regression equation
**speed**	**symmetry index**	0.29	0.023	y= 0.745 x + 0.198
**speed**	**most affected** **side**	0.24	0.055	y= 29.4 x + 20.3
**speed**	**less affected side**	-0.26	0.04	y= -31.7 x + 80.7

p value: Probability value; r: Pearson correlation coefficient.

### Children with hemiplegia

**Figure 3:**
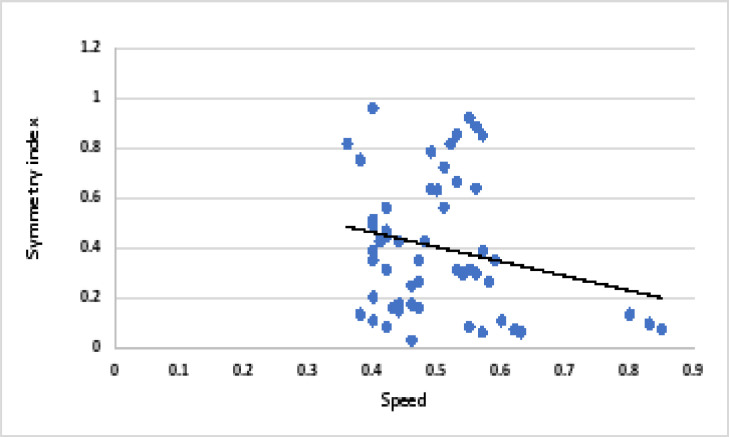
Correlation between speed and symmetry index in children with hemiplegia.

**Figure 4:**
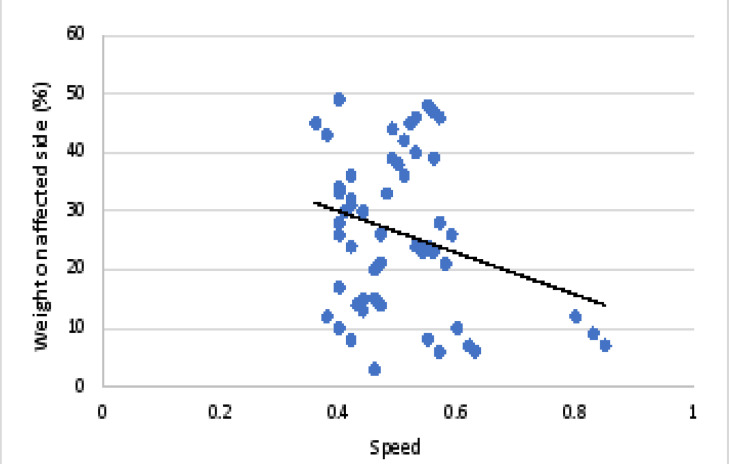
Correlation between speed and weight on affected side in children with hemiplegia.

There was a non-significant weak negative correlation between speed and symmetry index in children with hemiplegia. There was a significant weak negative correlation between weight on affected side and speed and a significant weak positive correlation between speed and weight on sound side. Correlation between speed and symmetry index in children with hemiplegia is represented in figure (3). Correlation between speed and weight on affected side in children with hemiplegia is represented in figure (4). Table (2) represents the correlation and regression equations for hemiplegia

**Table 2:** Correlation and regression equations for hemiplegia

		r value	p value	Regression equation
**speed**	**symmetry** **index**	-0.22	0.09	y= -0.579 x+ 0.690
**speed**	**affected side**	-0.27	0.04	y= -35.2 x + 43.7
**speed**	**sound side**	0.27	0.04	y= 34.8 x+ 56.5

p value: Probability value; r: Pearson correlation coefficient.

## Discussion

The objective of the study was to investigate the relation between the distribution of weight on both lower extremities and gait speed in children with spastic CP. For that, one hundred and fourteen children with spastic cerebral palsy (60 with diplegia, 54 with hemiplegia) was included in this study. Their gait pattern was evaluated by the Biodex gait trainer to record the distribution of body weight on both lower limbs and speed during gait.

The results revealed that, there was a significant weak positive correlation between speed and symmetry index in children with diplegia (r = 0.29, p = 0.023). Also, in diplegia there was a significant weak negative correlation between weight on less affected side and speed (r = -0.26, p = 0.04) and a nonsignificant weak positive correlation between speed and weight on most affected side (r = 0.24, p = 0.055).

The child with diplegia suffers high energy cost during walking as high as three to four times that of non-disabled children. High energy cost will cause a deterioration in walking. Co-contraction of the antagonists of the lower limbs lead to increase energy consumption and slow speed during walking 17,18.

Meanwhile, a non-significant weak negative correlation between speed and symmetry index in children with hemiplegia (r = -0.22, p = 0.09) was recorded. This means that, the more the symmetrical distribution of weight on both lower limbs, the less the speed will be. Significant weak negative correlation between weight on affected side and speed (r = -0.27, p = 0.04), and a significant weak positive correlation between speed and weight on sound side (r = 0.27, p = 0.04) was observed. This means that, the more the child bear weight on the sound limb, the more the speed will be and vice versa when loading the affected one. The child tends to underload the affected limb during the stance phase since bearing weight on the affected limb reduces his postural stability. To restore his stability, the child switches the weight from the affected to the sound limb rapidly, resulting in an increase in walking speed. This comes in agreement with the study by Domagalska-Szopa et al. 19, who confirmed that children with a tendency to underload the affected body side exhibit greater asymmetry in weight bearing and they have poor stability control in standing.

Although children with spastic hemiplegia have high functional levels and they are ambulatory, the weakness of muscles in the affected side results in functional impairments and affects the efficacy of walking relative to their peers 20. The child with hemiplegia suffers from asymmetry during performance of motor activities. Hemiplegic child prefers to bear his weight on the less affected lower limb which results in postural malalignments. These malalignments restrict his ability to transfer the weight to the affected lower limb 21.

A study by Elnaggar et al. 22, concluded that using an exercise to improve weight bearing symmetry on lower limb results in greater improvement of gait speed. Asymmetry of weight distribution is a common gait characteristic of children with CP which causes them to consume more energy and walk with less speed when compared with normal children of the same age 23. In the current study, children with diplegia and hemiplegia showed unequal weight distribution between both lower limbs which might affect the balance of the child during walking with a subsequent effect on the walking speed and leads to less-efficient walking performance. This is supported by the study by Woollacott and Shumway-Cook 24 who concluded that children with spastic diplegia and hemiplegia have reduced reactive postural control. The children need a longer time to regain their balance. Problems of coactivation of agonists and antagonist muscles, disorganized timing of muscle responses and delayed onset of muscle contractions contribute to reduced postural control and walking efficcy during unsupported locomotion.

Results of this study revealed that both children with diplegia and hemiplegia exhibit asymmetrical weight bearing on lower limbs. It was believed that asymmetric alignment in posture is especially characteristic in children with unilateral CP (spastic hemiplegia) 25. However asymmetrical behavior of the lower limbs is not characteristic only for hemiplegic population, so the authors hypothesized that children with CP present asymmetrical patterns of static balance and spatiotemporal gait characteristics and that asymmetry of the lower limbs load in a standing position may cause asymmetry of spatiotemporal gait parameters. To assess the level of symmetry the authors used the symmetry ratio proposed by Patterson et al., 26. For that, the selection of spastic diplegic children in addition to the spastic hemiplegic children was used to identify if other categories of CP children rather than the hemiplegic children present asymmetrical patterns of weight bearing on lower limbs. The results of this study showed that also the diplegic children has unequal weight distribution on both lower limbs as they prefer to increase load more on one side that affects speed of gait.

The most known variable to affect other gait kinematics is the speed of gait 27. Always, there is a change in walking speed after physical therapy treatment 28. Unfortunately, the effect of weight distribution asymmetry and speed of gait was not clearly investigated in the research studies of CP children. Studies on the adult suggested that increasing gait speed may result in decreased gait asymmetry 29,30. It is not the same in the healthy population^31^. In contrast, the asymmetries of gait in cerebral palsy children are increased when they run 32.

Slight differences are usually observed between limbs during normal gait even it is generally spatially and temporally symmetrical. Meanwhile, hemiplegic gait is generally asymmetrical as their gait pattern is characterized by biomechanical as well as spatial and temporal differences between the affected and less affected lower limbs. These temporal asymmetries may be observed during stance and swing phases 33. In the spatial asymmetry, the symmetry of step length is mainly affected in the children with hemiplegia 34,35. According to the findings of the study, children with spastic diplegia have a significant relationship between symmetrical weight bearing on both lower limbs and speed, whereas children with hemiplegia have a non-significant relationship.

## Limitations of the study

The sample size estimation for the proper participants involved in the study was sixty individuals for each group (hemiplegia, diplegia), however the available hemiplegic sample was only 54 children.

## Conclusion

Increasing the weight load on the affected side increases the speed of gait in hemiplegic children; while there is a less effect of increasing the load on the most affected side in diplegic children on the speed of gait. It is recommended to introduce exercises to improve the symmetrical loading on both lower extremities in children with cerebral palsy to improve gait speed and to promote efficient functional gait.

## Implication on physical therapy practice

Regaining the symmetry of weight bearing during gait is the correct way to improve walking speed in spastic cerebral palsy children, constructing a rehabilitation program to improve gait in those children including weight shifting and balance exercises could help achievement of this goal.
